# Simulating Cortical Feedback Modulation as Changes in Excitation and Inhibition in a Cortical Circuit Model

**DOI:** 10.1523/ENEURO.0208-16.2016

**Published:** 2016-08-31

**Authors:** Edward Zagha, John D. Murray, David A. McCormick

**Affiliations:** 1 Department of Neuroscience, Yale School of Medicine, New Haven, Connecticut 06520; 2 Department of Psychology, University of California Riverside, Riverside, California 92521; 3 Department of Psychiatry, Yale School of Medicine, New Haven, Connecticut 06520; 4 Kavli Institute for Neuroscience, Yale School of Medicine, New Haven, Connecticut 06520

**Keywords:** cortical circuit, cortical feedback, inhibition, neuromodulation, sensorimotor, top-down

## Abstract

Cortical feedback pathways are hypothesized to distribute context-dependent signals during flexible behavior. Recent experimental work has attempted to understand the mechanisms by which cortical feedback inputs modulate their target regions. Within the mouse whisker sensorimotor system, cortical feedback stimulation modulates spontaneous activity and sensory responsiveness, leading to enhanced sensory representations. However, the cellular mechanisms underlying these effects are currently unknown. In this study we use a simplified neural circuit model, which includes two recurrent excitatory populations and global inhibition, to simulate cortical modulation. First, we demonstrate how changes in the strengths of excitation and inhibition alter the input–output processing responses of our model. Second, we compare these responses with experimental findings from cortical feedback stimulation. Our analyses predict that enhanced inhibition underlies the changes in spontaneous and sensory evoked activity observed experimentally. More generally, these analyses provide a framework for relating cellular and synaptic properties to emergent circuit function and dynamic modulation.

## Significance Statement

Interactions with our surroundings are not fixed, but vary according to our internal goals and desires. By modulating the input–output properties of cortical circuits to match task demands, neuromodulation of the neocortex is essential for this behavioral flexibility. Experimental studies have demonstrated robust effects of neuromodulators on sensory responses, and a wide diversity of neuromodulatory actions on cellular targets. However, we still lack a robust framework for linking cellular properties to input–output properties of cortical circuits. To address this issue, we explore how a simplified circuit model can be modulated to produce a variety of functional circuits with different input–output properties. By comparing experimental and modeling data, we can make predictions about how cellular perturbations contribute to cortical modulations observed *in vivo*.

## Introduction

In the rodent whisker system, there is a prominent cortical feedback pathway from the whisker region of the motor cortex (MCtx) to the whisker representation in the primary somatosensory cortex (SCtx; Miyashita et al., 1994; Veinante and Deschênes, 2003). This pathway may be important for signaling movement-related and/or attentional-related activity (Lee et al., 2008; Petreanu et al., 2012; Zagha et al., 2013), analogous to the primate visual-oculomotor system (Rizzolatti et al., 1987; Moore et al., 2003; Cavanaugh et al., 2016). To explore the physiological impact of the MCtx–SCtx feedback pathway, recent studies have stimulated this pathway while recording network and cellular responses in the SCtx. Zagha et al. (2013) reported prominent modulation of SCtx network activity, including reduced spontaneous slow oscillatory activity and enhanced reliability of sensory representations. Other studies identified excitatory and inhibitory cellular targets of the MCtx feedback pathway (Rocco and Brumberg, 2007; Lee et al., 2008, 2013; Petreanu et al., 2009; Zagha et al., 2013; Kinnischtzke et al., 2014). However, despite these studies, we are still unable to identify which cellular mechanisms (excitatory/inhibitory interactions) underlie the network modulations (enhanced sensory representation).

A major impediment to translating between cellular and network function is that interactions between cortical neurons are recurrent and highly nonlinear. In such cases, computational models can provide intuitive descriptions of complex phenomena. In this study, our goal is to develop this intuition by implementing a cortical circuit model that is highly reduced, yet sufficiently complex to provide insights into cortical modulation. The circuit model we use was developed by Xiao-Jing Wang and colleagues, and has previously been used to model evidence accumulation and control of motor initiation in different cortical regions and species (Wong and Wang, 2006; Wong et al., 2007; Lo et al., 2009; Zagha et al., 2015). The model consists of two recurrent excitatory ensembles and a global inhibitory population (Wang, 2002). Instead of optimizing the model to simulate a specific neural process, here we demonstrate how the parameterization of this model can be used as a framework for the modulation of cortical function.

By changing the effective strengths of excitation and inhibition within the model, we examine the response properties of a reduced circuit model across a diversity of input–output processing states. Next, we provide inputs to the circuit model analogous to the sensory inputs delivered *in vivo*. By comparing the input–output properties of the models to the effects of cortical feedback modulation, we generate testable predictions about how cellular properties relate to cortical dynamics and sensory responsiveness. Ultimately, we predict that MCtx feedback modulations of SCtx network activity may be due to increases in the effective strength of inhibition and that increased inhibition underlies both reduced slow oscillatory activity and enhanced reliability and precision of sensory responses. Furthermore, we predict that changes in sensory response reliability and temporal precision are differentially related to the presence of intrinsic activity versus the integrative properties of the underlying circuit.

## Materials and Methods

### Circuit model

Wang (2002) and Wong and Wang (2006) have described the original spiking neuron network and mean-field reduction to a two-variable model. Here, we present a parameterization of the reduced circuit model, to simulate the modulatory effects of cortical feedback.

The model consists of two competing neural ensembles, which receive independent external inputs. The transfer function between total synaptic input (*I*) and firing rate (*r*) is as follows (Eq. 1):

$$r=f\left(I\right)=\frac{a\cdot I-b}{1-\exp\left[-d\left(a\cdot I-b\right)\right]}$$

Parameter values are *a* = 270 Hz/nA, *b* = 108 Hz, *d* = 0.154 s. The total synaptic inputs for each ensemble (*I1*, *I2*) are defined as follows:

I1=Js·S1−Jo·S2+Istim1+Inoise1

and

I2=Js·S2−Jo·S1+Istim2+Inoise2

Synaptic coupling parameters *Js* and *Jo* vary in our models (*Js*, 0.01:0.74 nA; *Jo*, 0.01:0.37 nA for full range and *Js*, 0.01:0.37 nA; *Jo*, 0.01:0.37 nA for transient response range). *Istim* varies according to simulation, from transient pulses to oscillatory waveforms or both. *Inoise* simulates background synaptic white noise [ni(t)] as follows (Eq. 2):

$$\tau noise\left[\frac{dInoise\left(t\right)}{dt}\right]=\;-(Inoise\left(t\right)-Io)+ni\left(t\right)\;\cdot \sqrt{\tau noise}\;\cdot \;\sigma noise$$

with noise amplitude σ*noise* varying from 0.001:0.02 nA and time constant τ*noise* = 10 ms. The amplitude of the background inputs (*Io*) varies for each simulation. *Inoise* was independent for each ensemble.

The network activity of each ensemble (*S*) is defined as follows (Eq. 3):

$$\frac{dS}{dt}=-\frac{S}{\tau s}+(1-S)\;\cdot \;\gamma \;\cdot \;r$$

with γ = 0.641 and τ*s* = 100 ms. This slower network time constant reflects the combination of fast (AMPA) and slower (NMDA) synaptic currents, and propagation time through an ensemble.

### Simulations

#### Coincident transient stimuli

Transient external stimuli were implemented as 10 ms duration step pulses of varying amplitude. Stimulus inputs were filtered with a 10 ms time constant, as described for the noise inputs above. For Figures 2 and 3, stimulus amplitude was 0.5 nA for ensemble 1 and varied from 0 to 2 nA for ensemble 2. For these simulations, noise parameters were as follows: *Io* = 0.32 nA; σ*noise* = 0.001 nA. For Figures 7 and 8, transient stimuli were of fixed proportion between ensembles (*Istim2* = 0.6 · *Istim1*); *Istim1* varied from 0.06 to 0.6 nA. For these simulations, noise parameters were as follows: *Io* = 0.32 nA; σ*noise* = 0.02 nA.

#### Sinusoidal inputs

Two hertz sinusoidal inputs were used to simulate a propagating slow oscillation. The amplitude of the sine wave was 0.05 nA (Figs. 6, 7). For these simulations, noise parameters were as follows: *Io* = 0.33 nA; σ*noise* = 0.001 nA.

Simulations used the full range of synaptic coupling parameters *Js* and *Jo* for Figures 2 and 3 and the transient response range of *Js* and *Jo* for Figures 6–8. Examples in Figure 2 have values as follows: *Js*: ***A***, 0.17; ***B***: 0.33; ***C***: 0.65; *Jo*: ***A***, 0.01; ***B***, 0.33; ***C***, 0.33. Examples in Figure 6 have values as follows: *Js*: ***A***, 0.35; ***B***: 0.35; ***C***: 0.43; ***D***: 0.01; *Jo*: ***A***, 0.01; ***B***, 0.01; ***C***, 0.01; ***D***; 0.35; *I*(*DC*): ***A***, 0.32; ***B***, 0.34; ***C***, 0.32; ***D***, 0.32. Examples in Figures 7 and 8 have values as follows: *Js*: mid-*Js*, 0.35; low *Js*, 0.01; *Jo*: low *Jo*: 0.01; high *Jo*: 0.35.

### *In vivo* recordings

Zagha et al. (2013) provide detailed descriptions of the recording, optogenetic, and whisker stimulation methods and analyses. Relevant experimental details are briefly discussed below. Three to five weeks before recording, ChR2 was expressed selectively in the MCtx by direct adeno-associated virus injection. Local field potential (LFP) and multiunit activity (MUA) recordings were obtained in mice sedated with chlorprothixene and anesthetized with urethane. All recording were targeted to layer V of the SCtx. Cortical feedback stimulation was paired with whisker deflection stimuli. Cortical feedback was stimulated by LED activation of ChR2 in the MCtx using slowly ramping illumination. Whisker stimuli were delivered by piezo-controlled whisker deflections of the principal whisker in the caudal direction. Deflections consisted of 5 ms ramps of varying amplitude, ranging from 140 to 1400°/s.

### Data analyses

Model simulations and analyses were conducted in Matlab (MathWorks). Multiunit spike times were determined as threshold crossings well isolated (>2× amplitude) from background noise. LFP was isolated by low-pass filtering off-line (100 Hz cutoff) and downsampling to 200 Hz. Power spectral densities were calculated using a multitaper method. Trial-by-trial (TbT) reliability was calculated as the mean correlation coefficient from all possible pairwise cross-correlation calculations of single-trial (LFP or *S*) responses. Input–output amplitude (I–O amp) and tau (I–O tau) were calculated as the average peak (I–O amp) and decay time constant (I–O tau) of the cross-correlation between stimulus (whisker or transient) input and single-trial (LFP or *S*) responses. The decay time constant was determined from an exponential fit of the average cross-correlagram, from peak to 100 ms lag. Single-trial correlation analyses were not conducted on MUA, due to the binary nature of spikes and strong dependence of correlation structure on spike rate. We did, however, calculate the average MUA time course for comparison to model data. To calculate the time to half-maximal response (T50) for MUA signals, we plotted the cumulative response profile from 10 ms post-stimulus (to account for sensory conduction delays) to 80 ms post-stimulus. This analysis was conducted on the average MUA response across all whisker stimulus intensities. For model data, the cumulative response profile was calculated from 0 to 100 ms post-stimulus across all transient stimuli. Data are presented as mean ± SE. Statistical testing was performed using paired Student’s *t* test (Table 1).

**Table 1.** Statistical tests and values

Graph	Type of test	Statistical values
a. Figure 5C	Paired *t* test, two-tailed	*t*_(6)_ = 3.99, *p* = 0.00716
b. Figure 5D	Paired *t* test, two-tailed	*t*_(6)_ = 4.24, *p* = 0.00542
c. Figure 5E	Paired *t* test, two-tailed	*t*_(6)_ = 4.00, *p* = 0.00711
d. Figure 5F	Paired *t* test, two-tailed	*t*_(6)_ = 3.84, *p* = 0.0086
e. Figure 5G	Paired *t* test, two-tailed	*t*_(6)_ = 6.06, *p* = 0.000914

## Results

### Description of the circuit model

**Figure 1.**
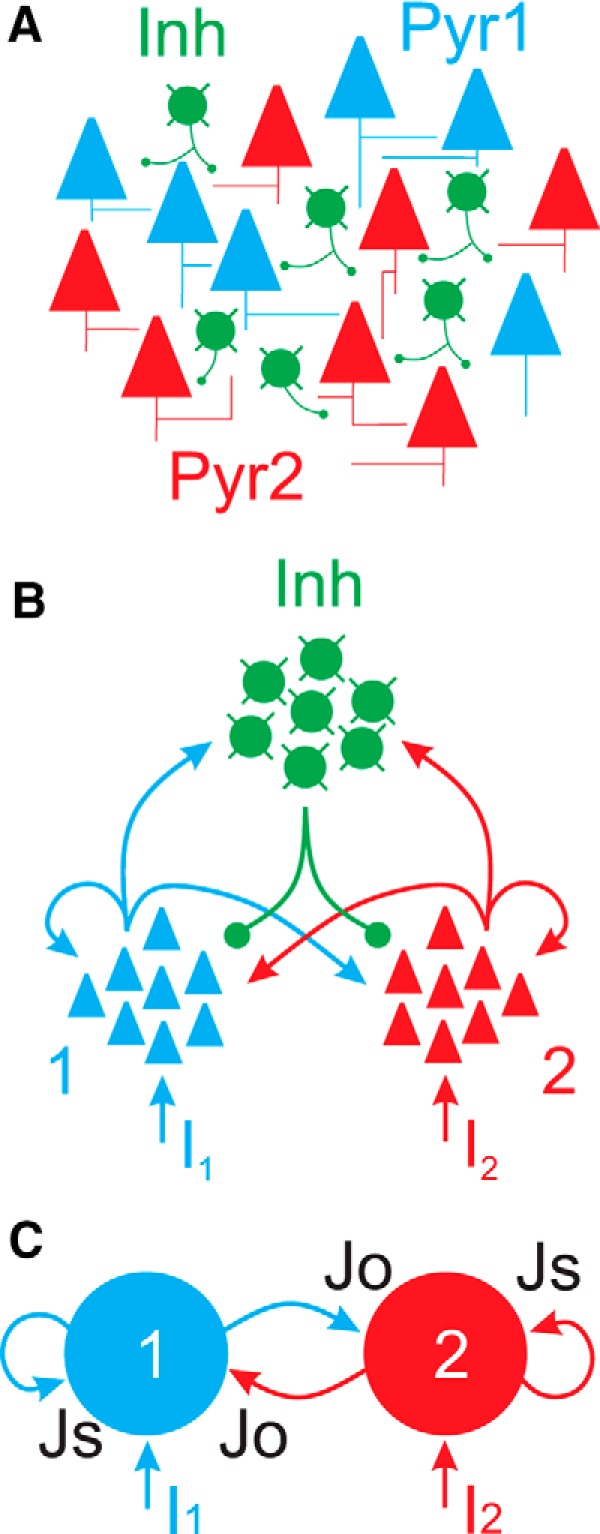
The circuit model. ***A***, The basic anatomical circuit consists of two populations of excitatory neurons (Pyr1, Pyr2) and a single population of inhibitory neurons (Inh). In cortical circuits, these populations may be intermingled, as depicted here. ***B***, By segregating the different populations, we highlight the major synaptic pathways. Each excitatory population projects to itself (recurrent) as well as the inhibitory and other excitatory populations. The inhibitory population receives inputs from, and projects to, both excitatory populations, providing global (within population and cross-population) inhibition. External inputs (*I1*, *I2*) are defined independently for each excitatory population. ***C***, The population representation was further reduced to two ensembles of excitatory neurons. Inhibition is encoded in the magnitude of synaptic coupling parameters *Js* (self) and *Jo* (other).

The original description of the circuit model was made by Wang (2002) and Wong and Wang (2006). Essential details of the model are summarized here. The circuit model consists of two populations of excitatory neurons and one population of inhibitory neurons (Fig. 1). Each excitatory population receives strong recurrent inputs, weak lateral excitatory inputs, and independent external inputs. Accordingly, each excitatory population can be considered as interconnected excitatory neurons with similar tuning to external stimuli, whereas different excitatory populations may be tuned to different stimulus features. The single inhibitory population mediates global (within population and cross-population) inhibition by integrating inputs from, and projecting to, both excitatory populations (Fig. 1*B*). The circuit model was further simplified by converting each excitatory population from spiking neurons to a single mean-field unit or ensemble (Wong and Wang, 2006; Fig. 1*C*). In this reduced model, synaptic parameter *Js* (self) refers to the combination of both self-excitation and within-ensemble (feedback) inhibition and synaptic parameter *Jo* (other) refers to the combination of both cross-excitation and cross-ensemble (lateral) inhibition. The models presented here are symmetric, such that *Js1* = *Js2* and *Jo1* = *Jo2*. Within-population interactions are net excitatory such that *Js* > 0, whereas cross-population interactions are net inhibitory such that *Jo* < 0. Accordingly, we refer to changes in *Js* as primarily changes in the strength of self-excitation and changes in *Jo* as primarily changes in the strength of cross-inhibition. In the figures and text, “Jo” refers to |Jo|, such that “high Jo” indicates strong cross-inhibition (*Jo* ≪ 0). In the simulations, these synaptic parameters are weighted by synaptic gating variable *S*, which reflects the activity level of each ensemble and is monotonically related to mean firing rate (see Materials and Methods). Time-varying inputs (*I1* and *I2*) into each ensemble incorporate both external inputs and background synaptic noise (see Materials and Methods).

### Diversity of input–output responses to transient, coincident stimuli

Wong and Wang reported that the operational mode of this circuit depends on the magnitudes of synaptic weights and external inputs (Wong and Wang, 2006, their Figs. 10, 12). Here, we extend these findings by explicitly parameterizing the net strengths of self-excitation (*Js*) and cross-inhibition (*Jo*). Also, we use external inputs that are designed to characterize input–output temporal dynamics and lateral suppression, and that are analogous to experimental stimuli described later in this study. With these simulations, we explore how the response behavior of these models change according to levels of self-excitation and cross-inhibition, specifically for repetitive, transient stimuli.

**Figure 2.**
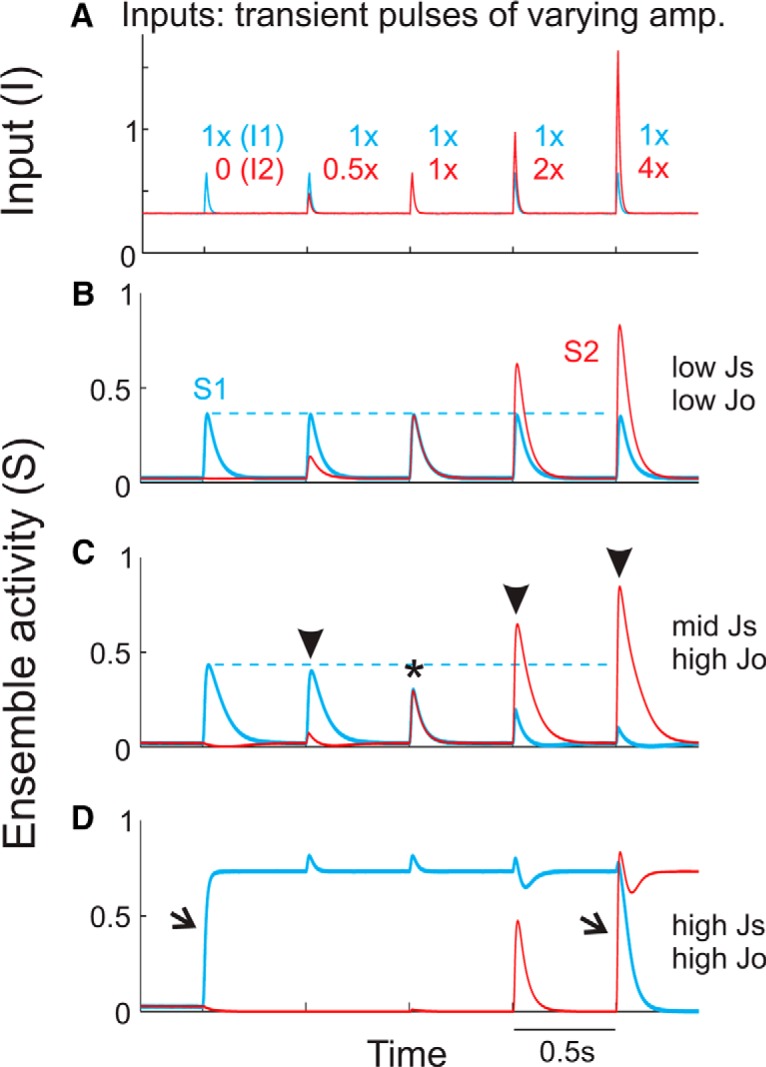
Diverse circuit model responses to transient, coincident stimuli. ***A***, External inputs to each ensemble (*I1*, *I2*) consisted of transient, coincidence current pulses, on top of background synaptic activity (see Materials and Methods). Transient inputs to ensemble 1 (blue) were of fixed amplitude, whereas transient inputs to ensemble 2 (red) increased from 0 to 4 × *I1*. ***B--D***, Ensemble activity levels (*S1*, *S2*) in response to the above inputs, for various values of *Js* and *Jo*. ***B***, For models with low *Js* and low *Jo*, network responses are transient and largely independent. ***C***, For models with mid-*Js* and high *Jo*, network responses are transient, yet with strong cross-suppression. The asterisk depicts functionally equal cross-suppression, whereas the arrowheads depict functionally asymmetric cross-suppression, in which the ensemble with higher activity is preferentially suppressing the ensemble with lower activity. ***D***, For models with high *Js* and high *Jo*, network responses are sustained with extreme cross-suppression. Arrows depict instances in which transient inputs produced sustained, winner-take-all transitions.

We present a series of simultaneous, transient inputs to each ensemble (Fig. 2*A*). While ensemble 1 inputs were of fixed amplitude, ensemble 2 inputs varied from 0 to 4× ensemble 1 inputs. We consider this analogous to presenting one oriented stimulus at a fixed contrast/intensity and a second stimulus with a different orientation at increasing contrast/intensity. With low self-excitation (*Js*) and low cross-inhibition (*Jo*), ensemble responses were transient and largely independent of each other (Fig. 2*B*). With mid-level self-excitation and high cross-inhibition (Fig. 2*C*), responses were again transient, with prolonging of transient responses in models with increased self-excitation. The elevated cross-inhibition, however, yielded strong cross-ensemble suppression. Suppression could be in the form of cosuppression with equal weights for similar inputs (Fig. 2*C*, asterisk) or nonlinear increases in cross-ensemble suppression for dissimilar inputs (Fig. 2*C*, arrowheads). This pattern of cross-suppression is similar to experimental findings of lateral suppression within multiple sensory cortical areas (Busse et al., 2009; Carandini and Heeger, 2012). For models with high self-excitation and high cross-inhibition (Fig. 2*D*), transient stimuli were converted to sustained, winner-take-all responses (arrows). These responses are characterized by the generation of persistent activity within an ensemble that outlasts the stimulus and suppresses responses in the nonpersistently discharging ensemble. Given sufficient stimulation of the inactive ensemble, it can become persistently active and suppress the activity in the previously active ensemble (Fig. 2*D*). This winner-take-all activity is reminiscent of models of sustained responses in prefrontal and premotor cortices during working memory or motor planning (Miller et al., 2003; Constantinidis and Wang, 2004).

**Figure 3.**
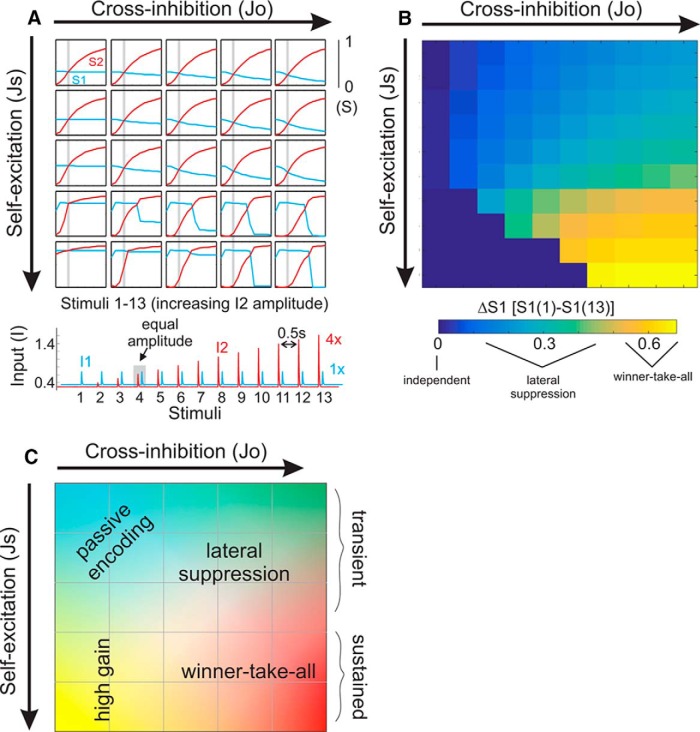
Patterns of cross-suppression across a wide range of values for synaptic coupling parameters *Js* and *Jo*. *A*, Top, Plot of peak responses of both ensembles for each of the transient stimuli. The trajectories of *S2* reflect both increasing stimulus amplitude and intrinsic circuit dynamics. As the transient inputs to ensemble 1 (*I1*) are equivalent throughout this simulation, differences in *S1* trajectories reflect changes in circuit dynamics alone. The gray bar in each plot refers to the condition in which *I1* = *I2*. For some simulations, we find that the *S1* and *S2* plots intersect at this point. For other simulations, the point of intersection is shifted rightward, indicating a dependence on previous activity. Bottom, Extended stimulus series used to generate the above data. ***B***, Quantification of cross-suppression, as the difference in *S1* from the first compared to the last stimulus (Δ*S1*). ***C***, Illustration summarizing the functional regimes accessible through parameterization of our circuit model. Changing self-excitation and cross-inhibition produces a family of functional circuits with diverse input–output response properties. Indication of “transient” versus “sustained” regimes are based on simulations as shown in Figure 2.

To further explore the range of responses produced by these circuit models, we systematically parametrized *Js* and *Jo*, while presenting an extended series of stimuli (13 stimuli with fixed *I1* amplitude to ensemble 1 and increasing *I2* amplitude to ensemble 2; Fig. 3*A*, bottom). In Figure 3*A* (top), we plot the peak response of each ensemble (*S1* and *S2*) to each of the 13 stimuli. One interesting difference across this parameter space is the dependence of ensemble 1 on ensemble 2 activity. In Figure 3*B*, we quantify this dependence as the difference in *S1* (Δ*S1*) comparing the first (in which *I2* = 0) and last (*I2* = 4 × *I1*) stimulus responses. For models with low cross-inhibition Δ*S1* was near 0, reflecting *S1* responses that were very similar in amplitude and therefore largely independent of *S2*. For models with higher cross-inhibition and moderate self-excitation, we observed intermediate levels of Δ*S1* (0.2–0.5). Note that for these models, *S1* decreases continuously with increasing intensity of *I2* (and *S2*; Fig. 3*A*). Furthermore, we observe *S1–S2* crossing points at stimulus 4, in which *I1* = *I2* (Fig. 3*A*, gray bars). For models with high cross-inhibition and high self-excitation, we observed abrupt, winner-take-all transitions, from near maximal to near minimal activity levels. This is reflected in large Δ*S1* values (>0.5). Furthermore, in these models, the *S1–S2* crossing points are right-shifted, at conditions in which *I2* > *I1*. As these models display sustained responses to stimuli (Fig. 2*D*), these dynamics reflect a strong dependence on past events and robustness against competing inputs.

Overall, we find that by tuning a small number of parameters in a reduced circuit model, we are able to produce a wide diversity of input–output response dynamics. Across this parameter space, model circuits differ in their levels of passive encoding of external inputs, lateral suppression, and winner-take-all and high-gain states (Fig. 3*C*). Moreover, the same circuit can produce transient (low *Js*) or sustained (high *Js*) responses to stimuli.

Now that we have defined the input–output landscape of the circuit for transient stimuli, we can use this landscape as a reference for predicting relationships between changes in synaptic weights and input–output response properties. To do this, we first analyze the effects of cortical feedback modulation on transient sensory responses *in vivo*. Next, we determine the changes in synaptic weights of the model that are sufficient to simulate the cortical response modulations observed experimentally.

### Stimulus–response population dynamics and modulation *in vivo*

**Figure 4.**
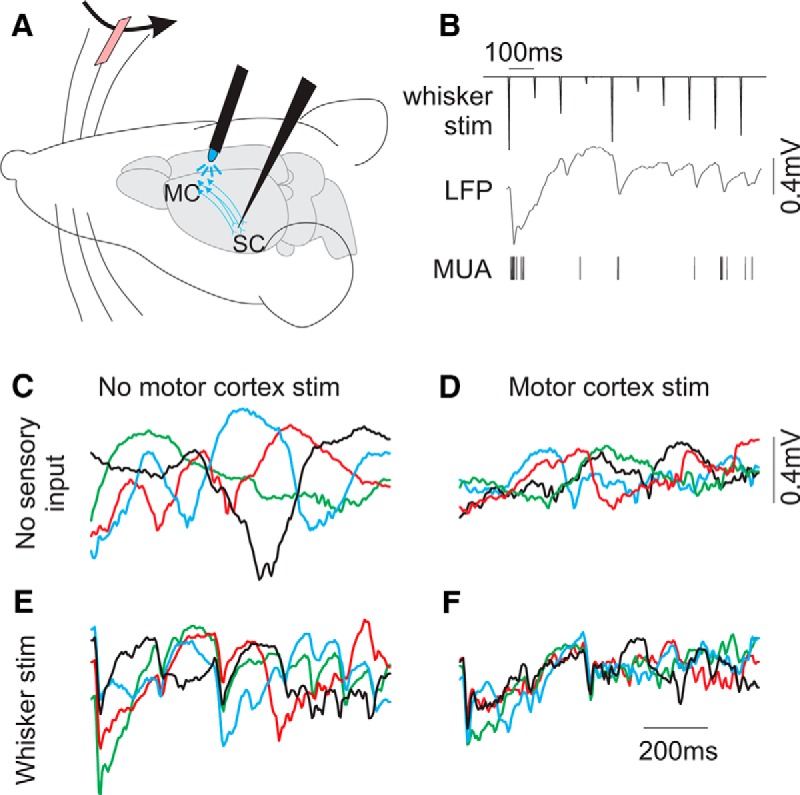
Probing the effects of top-down cortical feedback on sensory responses. ***A***, Schematic of the experimental design. ChR2 was expressed in MCtx neurons, and stimulated by light application in the MCtx. The neurons in blue depict the cortical feedback pathway. In the contralateral whisker field, a single whisker was deflected by a piezo-controlled paddle. Cortical feedback and whisker sensory responses were recorded in primary SCtx. ***B***, Top, Example of a whisker stimulus pattern, consisting of a series of rapid deflections of varying amplitude. Bottom, Example single-trial population activity recorded in SCtx, in response to the above stimulus. ***C–F***, LFP signals overlaid from four trials of each condition: spontaneous activity (***C***), MCtx stimulation alone (***D***), whisker stimulation alone (***E***), and MCtx and whisker stimulation combined (***F***). Note that during MCtx stimulation, LFP signals in the SCtx show reduced-amplitude low-frequency activity (compare ***C***, ***D***). When paired with whisker stimulation, MCtx stimulation results in reduced in TbT variability, reduction of slow (>100 ms) dynamics, and increased resemblance to the whisker stimulus inputs (compare ***E***, ***F***).

**Figure 5.**
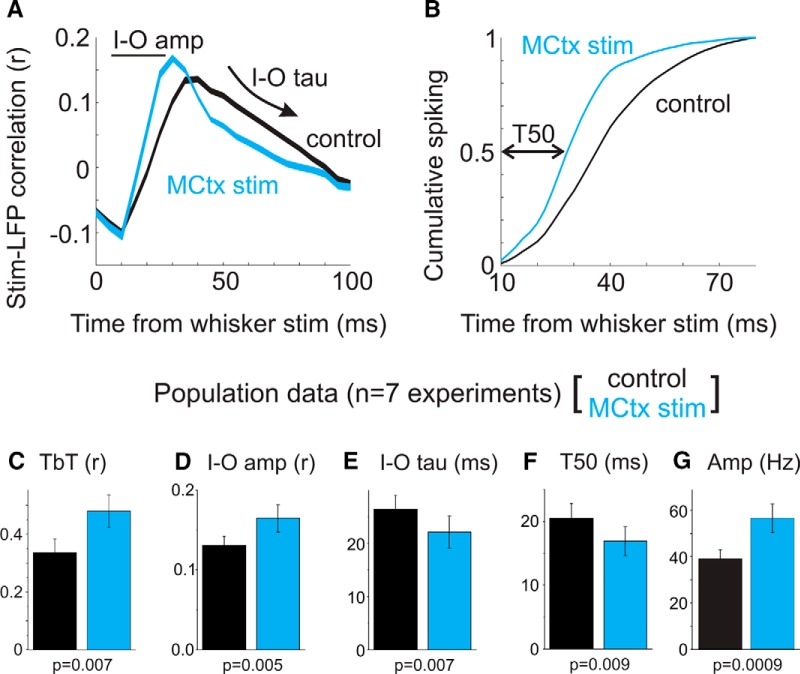
Quantification of sensory response modulation by cortical feedback activation. ***A***, For LFP signals, single-trial responses were cross-correlated with the whisker stimulus input. Shown here is the average cross-correlation function from one recording session, for conditions of whisker stimulation alone (control, black) and combined MCtx and whisker stimulation (MCtx stim, blue). Input–output amplitude (I–O amp) and tau (I–O tau) refer to the peak correlation amplitude and decay, respectively. ***B***, For MUA signals, we plotted the normalized, cumulative spiking aligned to all transient whisker stimuli, and determined T50. The 10 ms offset accounts for sensory conduction delays. ***C–G***, Population data for measurements of sensory response reliability (***C***, ***D***), temporal precision (***E***, ***F***), and response amplitude (***G***; black, control; blue, MCtx stim).

In anesthetized mice, population activity (LFP and MUA) was recorded in layer V of the SCtx (Zagha et al., 2013). MCtx neurons were optogenetically stimulated, thereby activating the motor-sensory feedback pathway (Fig. 4). Continuous, ramping illumination onto the MCtx was applied for 2–5 s. As described by Zagha et al. (2013), MCtx stimulation strongly influenced SCtx spontaneous and sensory-evoked activity. MCtx stimulation caused rapid SCtx activation, characterized by a marked suppression of low-frequency (1–4 Hz) population activity and enhancement of gamma-band (30–50 Hz) activity (Fig. 4*C*,*D*). Additionally, rapid (10 ms) caudal deflections of the principal whisker were paired with MCtx stimulation. As previously reported (Zagha et al., 2013), cortical feedback stimulation increased TbT reliability of LFP signals (43% increase; Figs. 4*E*,*F*, 5*C*) and increased MUA response amplitude (44% increase; Fig. 5*G*).

Here, we report additional analyses of this experimental data on the sensory-response properties of the SCtx in the absence (control) or presence of MCtx stimulation (Fig. 5). By cross-correlating the inputs (whisker stimulus patterns) and population (single-trial LFP) responses, we determined the cross-correlation peak amplitudes (I–O amp) and decay time constants (I–O tau; Fig. 5*A*). For MUA signals, we calculated the average response time course across all stimulus amplitudes, and determined the time to half-maximal response (T50, Fig. 5*B*). MCtx stimulation caused significant changes in each of these measurements (*n* = 7 recordings from *n* = 6 mice; Fig. 5*D–F*). For LFP signals, MCtx stimulation resulted in an increase in the input–output correlation amplitude [I–O amp (*r*), control: 0.13 ± 0.01; MCtx stimulation: 0.16 ± 0.02, *p* = 0.005] and decrease in the cross-correlation decay time constant (I–O tau: control, 26.4 ± 2.6 ms; MCtx stimulation, 22.1 ± 3.0 ms, *p* = 0.007). For MUA signals, MCtx stimulation decreased T50 (control, 20.5 ± 2.4 ms; MCtx stimulation, 16.9 ± 2.3 ms, *p* = 0.009). In summary, we find that MCtx stimulation reduces slow oscillatory activity and increases the input–output correlation and temporal precision of sensory signals in the SCtx.

### Circuit model responses to slow (spontaneous-like) and rapid (sensory-like) inputs

**Figure 6.**
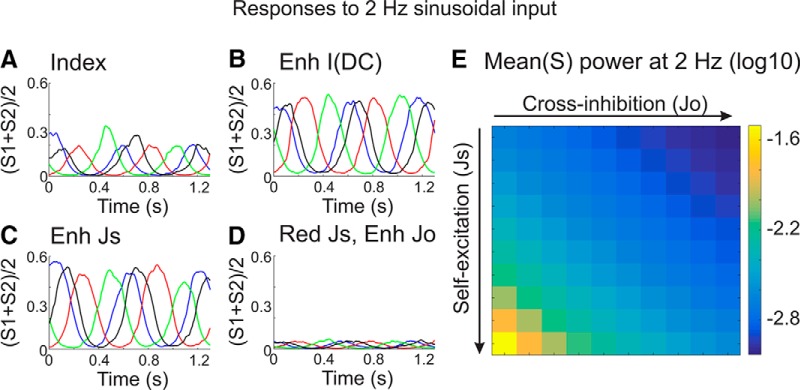
Diverse circuit model responses to slow oscillatory stimuli. ***A–D***, Average ensemble activity [(*S1* + *S2*)/2] in response to 2 Hz sinusoidal inputs. Equivalent sinusoidal inputs were applied to both ensembles. Each panel shows overlapping responses to four trials, in which the phase of the sine wave inputs differed on each trial. Note that different circuit models differentially amplify the same sinusoidal inputs. Simulations (***B–D***) refer to differences in model parameters compared to ***A***. ***B***, Increased background synaptic activity *I*(*DC*). ***C***, Increased self-excitation (*Js*). ***D***, reduced self-excitation (*Js*) and enhanced cross-inhibition (*Jo*). ***E***, 2 Hz power in the average ensemble activity, from models with varying ***Js*** and ***Jo***, in response to 2 Hz sinusoidal inputs.

Cortical circuits under anesthesia and during quiet wakefulness produce spontaneous, low-frequency (0.1–5 Hz) oscillations (i.e., the slow oscillation). Our circuit model does not generate this spontaneous rhythmic activity. Therefore, we simulate conditions in which the local circuit receives a slow oscillatory external input, and study how the circuit models propagate such activity. We simulate the slow oscillation as a common 2 Hz sine wave-modulated input to each ensemble (Fig. 6). Note that in these simulations, we explore only the “transient” range of parameter space (low to mid-*Js*, full-range *Jo*; Fig. 3), since the *in vivo* activity we are simulating displayed transient responses to rapid inputs before and after modulation. Furthermore, instead of resolving *S1* and *S2* independently, here we analyze the average population activity [(*S1* + *S2*)/2], analogous to the LFP and MUA measurements described above.

The example circuit model shown in Figure 6*A* responds to the sinusoidal input with a prominent sinusoidal output. How may a modulatory input reduce the propagation of these slow signals in our circuit models? Adding a DC input (increasing *I1* and *I2*) or increasing self-excitation (increasing *Js*), actually increases the sinusoidal output of our circuit (Fig. 6*B*,*C*). Alternatively, reducing self-excitation and/or increasing cross-inhibition reduces the sinusoidal response amplitude (Fig. 6*D*). We calculated the 2 Hz power in the output of each model, in response to the same sinusoidal input (Fig. 6*E*). According to our circuit model, an effective mechanism to reduce the propagation of the slow oscillation would be to reduce self-excitation and/or enhance cross-inhibition.

**Figure 7.**
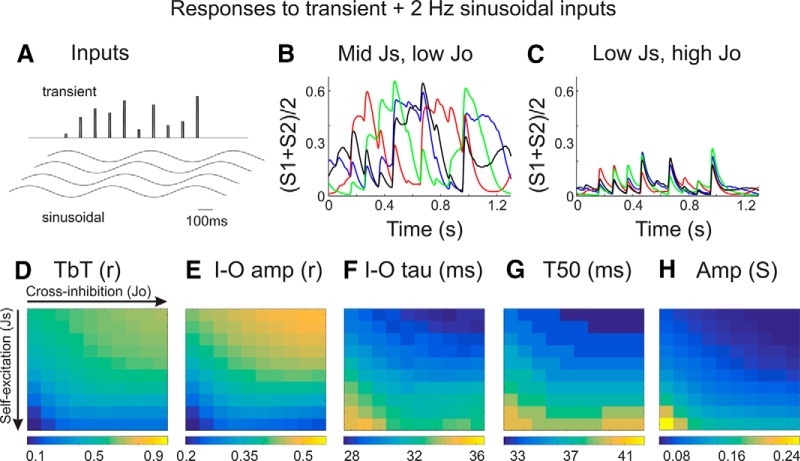
Circuit model responses to slow (spontaneous-like) and rapid (sensory-like) inputs. ***A***, Transient and sinusoidal inputs used in these simulations. The two ensembles received identical sinusoidal inputs and proportional transient inputs (see Materials and Methods). Each trial began with a different phase of the sinusoidal input. ***B***, ***C***, Examples of average ensemble activities ([*S1* + *S2*]/2) overlaid from four different trials. Notice that the responses of the model in ***C*** appear to have higher TbT reliability, reduced slow (>100 ms) dynamics, and greater resemblance to the transient inputs. ***D–H***, Quantification of input–output response reliability (***D***, ***E***), temporal precision (***F***, ***G***), and response amplitude (***H***) from models with varying *Js* and *Jo*. Highest reliability (TbT r and I-O amp) and most rapid dynamics (I–O tau and T50) are observed in models with low self-excitation and high cross-inhibition.

To simulate sensory inputs, we stimulated each ensemble with simultaneous, transient pulses of proportional amplitude (Fig. 7*A*). We consider this analogous to presenting a single, oriented stimulus of varying contrast/intensity, as in the *in vivo* experiments described above. As the two ensembles are defined by having different stimulus feature preferences, we implement the stimuli as exciting one ensemble stronger than the other at all intensities, and therefore the transient inputs maintain a fixed proportion (*I2* = 0.6 × *I1*). We present multiple amplitudes to ensure that we sample across a wide range of responses. Furthermore, we present the transient inputs in the presence of the low-frequency (2 Hz) sinusoidal background.

For models with mid self-excitation and low cross-inhibition, there is significant TbT variability, presumably due to variability in the phase of the low-frequency oscillation on each trial (Fig. 7*B*). Reducing self-excitation and/or increasing cross-inhibition reduced TbT variability and appeared to increase the temporal precision of the transient responses (Fig. 7*C*). For each model, we calculated the TbT reliability, I–O amp, I–O tau, T50, and response amplitude (Fig. 7 *D–H*). The sensory responses with the highest fidelity and precision (high TbT reliability and I–O & low I–O tau and T50) occur in models with low self-excitation and high cross-inhibition (Fig. 7*D–G*).

According to the above analyses, the effects of cortical feedback modulation on slow oscillatory activity and sensory responses can be simulated as increases in inhibition within our circuit model; MCtx stimulation and increased circuit model inhibition similarly increased input–output reliability and temporal precision. One potential discrepancy, however, is response amplitude. While MCtx feedback stimulation significantly increased MUA response amplitude (Fig. 5*G*), increased inhibition in the model results in reduced activity (Fig. 7*H*). However, our experimental measure of MUA reflects both excitatory and inhibitory neuronal spiking, whereas the model variable *S* reflects only excitatory ensemble activity. Further considerations regarding response amplitude are presented in the Discussion.

### Influences of synaptic weights on sensory responses in the absence of the slow oscillation

In this section, we explore the inter-relationships between synaptic weights, the slow oscillation, and sensory responses. One prominent hypothesis is that neuromodulation-mediated enhanced sensory representations are direct consequences of reduced slow oscillations, since the slow oscillation is an intrinsic signal that competes with the neural representation of external events (Arieli et al., 1996). A second hypothesis, however, is that reduced slow oscillations and enhanced sensory representations are both consequences of changes in the integrative properties of the underlying neural circuit. While we do not have the ability to isolate these interdependent components *in vivo*, we can dissect them in the circuit model, as described below.

**Figure 8.**
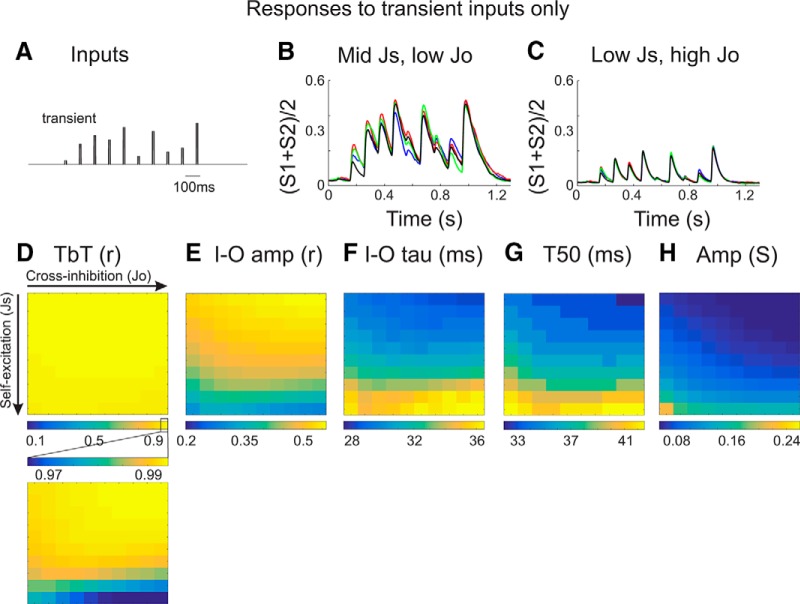
Circuit model responses to rapid (sensory-like) inputs alone. ***A***, Inputs used in these simulations, consisting of the transient inputs only. ***B***, ***C***, Examples of average ensemble activities [(*S1* + *S2*)/2] overlaid from four different trials. Notice the highly reduced TbT variability compared to traces in Figure 7*B*,*C*. ***D–H***, Quantification of input–output response reliability (***D***, ***E***), temporal precision (***F***, ***G***), and response amplitude (***H***), from models with varying *Js* and *Jo*. Scale bars in ***D–H*** are the same as scale bars in Figure 7***D–H***. Removing the sinusoidal inputs results in near-maximal TbT reliability and elevated I–O amp for all models (compare Figs. 7*D*,*E*, 8*D*,*E*). However, removing sinusoidal inputs alone does not account for increased temporal precision (compare Figs. 7*F*,*G*, 8*F*,*G*). The bottom plot in ***D*** depicts TbT reliability measurements, rescaled to show the dynamic range for this set of simulations. Even though correlation values are high (>0.95) for all models, synaptic weights still do modulate TbT reliability in this restricted range in the absence of the sine-wave inputs.

According to the first hypothesis, eliminating the slow oscillation (without changing the underlying circuit) should enhance sensory responses as effectively as changing the synaptic weights of the model. To test this, we presented the sensory inputs to each model, but now without the low-frequency sinusoidal background (Fig. 8). Elimination of the low-frequency inputs dramatically increased reliability measurements across all models, but had little influence on temporal dynamics. Without the slow oscillation, TbT reliability and I–O correlation amplitude were increased (averaged across all models: TbT reliability (*r*), 0.49 → 0.98; I–O amp (*r*), 0.38 → 0.48; Fig. 8*D*,*E*), consistent with the effects of cortical feedback on response reliability. Without the slow oscillation, temporal dynamics were slightly *prolonged* (averaged across all models: I–O tau, 30.4 → 30.7 ms; T50, 35.5 → 35.8 ms; Fig. 8*F*,*G*), which is in the opposite direction of the effects of cortical feedback on response dynamics. Extrapolating to our *in vivo* observations, our models predict that elimination of the slow oscillation alone is only partially responsible for the MCtx feedback modulation of sensory responses. In particular, changes in temporal dynamics may be independent of the presence of the slow oscillation and reflect changes in the excitatory/inhibitory structure of the underlying circuit.

## Discussion

### General findings

The goal of this study is to develop a set of computational and experimental data analyses to assess circuit mechanisms of cortical feedback modulation. We determined how various strengths of excitation and inhibition within a cortical circuit model affect sensory responsiveness, and then use this modeling data to predict the mechanisms underlying modulations of sensory responses observed experimentally. Using a mean-field reduced circuit model and tuning self-excitation and cross-inhibition, we could produce a family of circuits that differ widely in terms of sustained versus transient activity, lateral suppression, and sensory response fidelity. Next, we simulated experimental conditions in our circuit model, and performed the same input–output response analyses on the computational and experimental datasets. The main predictions from this study are as follows: (1) reduced propagation of the slow oscillation and increased reliability and temporal precision of sensory responses are primarily due to cortical feedback enhancement of local circuit inhibition; and (2) increases in temporal precision are directly related to increased intracircuit inhibition, whereas increases in response reliability are largely related to suppression of the slow oscillation.

### Interpretations and limitations of these circuit models

The circuit models used in this study are highly reduced, and delineate only the most basic scaffolding of known excitatory and inhibitory cortical circuits. And yet these models are surprisingly robust, as demonstrated by their ability to simulate a wide diversity of cortical processes (Wong and Wang, 2006; Wong et al., 2007; Lo et al., 2009; Zagha et al., 2015; Fig. 2, 3). A possible explanation is that the parameters described here, self-excitation and cross-inhibition, reflect the net effects of numerous cellular processes. Neuromodulators in neocortex are known to affect multiple cell types with diverse contributions to cortical circuits, through multiple metabotropic and ionotropic processes [e.g., (McCormick et al., 1993; Gu, 2002)]. Many of these effects can be modeled, in part, as changes in self-excitation or cross-inhibition. For example, reduced pyramidal cell excitability by activation of K+ currents (Andrade et al., 1986) can be partially represented as reduced self-excitation; pyramidal cell disinhibition by stimulation of inhibitory neuron-targeting interneurons (Lee et al., 2013) can be partially represented as increased self-excitation. While this interpretation enables multiple types of perturbations to be studied in these models, it also means that there is no unique mapping between the model parameter and the cellular mechanism.

Other processes, however, are not represented in these models. For example, these models lack intrinsic rhythmic activity and have coincident actions of excitation and inhibition, thereby potentially masking important roles of synchrony and oscillations. Also, subcortical structures are neglected in these models, which are known to powerfully influence cortical dynamics (Steriade et al., 1993; Poulet et al., 2012). These models can be expanded to simulate additional features of cortical and subcortical circuits, yet at the expense of increasing the number of parameters.

### Testing the predictions of this study

By comparing the effects cortical feedback stimulation with modeling data, we predict that increases in the effectiveness of intra-circuit inhibition most strongly underlie our experimental observations of enhanced sensory reliability and temporal precision. Does cortical feedback stimulation increase inhibitory activity? A previous *in vitro* study found that MCtx feedback stimulations depolarize both somatostatin and fast-spiking interneurons in layer V of the SCtx (Kinnischtzke et al., 2014). The *in vivo* experimental data analyzed in this study found increases in layer V MUA, which does not distinguish between cell types. Future studies need to determine the cell type-specific spiking response to MCtx feedback stimulation *in vivo*. We mentioned in the Results the apparent discrepancy between increased MUA in experimental data and predicted decreased activity (*S*) in the model. As variable *S* refers to activity of excitatory neurons only, we predict that the increased MUA may be due to significant increases in the spiking of interneurons, amongst decreases in the spiking of pyramidal cells. To directly observe changes in synaptic inhibition, additional studies are needed to record excitatory and inhibitory responses in pyramidal neurons. We predict that MCtx stimulation results in significantly increased inhibition and reduced or unchanged excitation. Moreover, it is quite possible that different circuit mechanisms dominate in other cortical layers and in other behavioral states (Lee et al., 2008, 2013).

Interestingly, recent experimental studies in other cortical areas have reported dominant roles for the modulation of inhibition within cortical circuits. In the visual cortex, wakefulness compared to anesthesia was associated with substantial increases in nonselective inhibition, reducing the spatial and temporal extent of sensory responses (Haider et al., 2013); in the auditory cortex, movement-related signals from the frontal cortex activated inhibitory neurons, reducing the amplitude of sensory responses from nearby excitatory neurons (Schneider et al., 2014). Inhibition is well known to be a major determinant of sensory responses, and has been studied as such in many brain regions and species [e.g., (Sillito, 1975; Perez-Orive et al., 2002; Cruikshank et al., 2007; Wimmer et al., 2015)]. These recent studies suggest that modulation of inhibition may be a general mechanism of sensory cortices to evoke rapid, context-dependent shifts in sensory responsiveness.

## Synthesis

The decision was a result of the Reviewing Editor Marlene Bartos and the peer reviewers coming together and discussing their recommendations until a consensus was reached. A fact-based synthesis statement explaining their decision and outlining what is needed to prepare a revision is listed below.
